# Supplementary material for: Exploring the attitudes of medical faculty members and students in Pakistan towards plagiarism: a cross sectional survey
Source: PeerJ. 2015 Jun 18;3:e1031. doi: 10.7717/peerj.1031 (PMC4476128; doi:10.7717/peerj.1031)
Supplement: Table S1 [file peerj-03-1031-s001.docx]

Table 4: Attitudes towards plagiarism of medical students and faculty members

| Statements of the ATPQ | Opinion | Frequency of responses | |
| --- | --- | --- | --- |
|  |  | Students | Faculty |
| Sometimes one cannot avoid using other people’s words without citing the source, because there are only so many ways to describe something. | Agree | 53.0% | 57.8% |
|  | Disagree | 27.3% | 32.2% |
|  | Neutral | 19.7% | 10.0% |
| It is justified to use previous descriptions of a method, because the method itself remains the same. | Agree | 51% | 52.2% |
|  | Disagree | 23.3% | 32.2% |
|  | Neutral | 25.7% | 15.6% |
| Self-plagiarism is not punishable because it is not harmful (one cannot steal from oneself). | Agree | 64.4% | 38.9% |
|  | Disagree | 21.6% | 42.2% |
|  | Neutral | 14.0% | 18.9% |
| Plagiarized parts of a paper may be ignored if the paper is of great scientific value. | Agree | 32.8% | 30.4% |
|  | Disagree | 52.5% | 53.9% |
|  | Neutral | 14.7% | 15.7% |
| Self-plagiarism should not be punishable in the same way as plagiarism is | Agree | 66.1% | 47.8% |
|  | Disagree | 20.4% | 34.4% |
|  | Neutral | 13.5% | 17.8% |
| Young researchers who are just learning the ropes should receive milder punishment for plagiarism. | Agree | 48.0% | 42.2% |
|  | Disagree | 36.8% | 46.7% |
|  | Neutral | 15.2% | 11.1% |
| I could not write a scientific paper without plagiarizing. | Agree | 25.4% | 22.2% |
|  | Disagree | 58.9% | 70.0% |
|  | Neutral | 15.7% | 7.8% |
| Short deadlines give me the right to plagiarize a bit. | Agree | 33.7% | 25.6% |
|  | Disagree | 49.7% | 62.2% |
|  | Neutral | 16.6% | 12.2% |
| It is justified to use one’s own previously published work without providing citation in order to completethe current work. | Agree | 42.7% | 27.8% |
|  | Disagree | 37.3% | 61.1% |
|  | Neutral | 20.0% | 11.1% |
| If a colleague of mine allows me to copy from her/his paper, I’m NOT doing anything bad, because I have his/her permission. | Agree | 49.4% | 28.9% |
|  | Disagree | 35.4% | 56.7% |
|  | Neutral | 15.2% | 14.4% |
| A plagiarized paper does no harm science | Agree | 33.0% | 24.4% |
|  | Disagree | 49.4% | 68.9% |
|  | Neutral | 17.6% | 6.7% |
| Since plagiarism is taking other people’s words rather than tangible assets; it should NOT be considered as a serious offense. | Agree | 17.1% | 11.1% |
|  | Disagree | 72.0% | 78.9% |
|  | Neutral | 10.9% | 10% |
| Authors say they do NOT plagiarize, when in fact they do | Agree | 56.5% | 72.3% |
|  | Disagree | 22.6% | 14.4% |
|  | Neutral | 20.9% | 13.3% |
| Those who say they have never plagiarized are lying. | Agree | 48.0% | 54.4% |
|  | Disagree | 28.0% | 27.8% |
|  | Neutral | 24.0% | 17.8% |
| Sometimes I’m tempted to plagiarize, because everyone else is doing it (students, researchers, physicians). | Agree | 43.4% | 37.8% |
|  | Disagree | 41.6% | 48.9% |
|  | Neutral | 15.0% | 13.3% |
| I keep plagiarizing because I haven’t been caught yet. | Agree | 17.3% | 10.0% |
|  | Disagree | 71.1% | 75.6% |
|  | Neutral | 11.6% | 14.4% |
| I work (study) in a plagiarism-free environment. | Agree | 20.4% | 24.4% |
|  | Disagree | 66.8% | 61.2% |
|  | Neutral | 12.8% | 14.4% |
| Plagiarism is not a big deal. | Agree | 20.2% | 13.3% |
|  | Disagree | 67.2% | 75.6% |
|  | Neutral | 12.6% | 11.1% |
| Sometimes I copy a sentence or two just to become inspired for further writing. | Agree | 51.3% | 48.9% |
|  | Disagree | 35.9% | 41.1% |
|  | Neutral | 12.8% | 10.0% |
| I don’t feel guilty for copying verbatim a sentence or two from my previous papers. | Agree | 46.8% | 36.7% |
|  | Disagree | 39.9% | 52.2% |
|  | Neutral | 13.3% | 11.1% |
| Plagiarism is justified if I currently have more important obligations or tasks to do. | Agree | 25.9% | 17.8% |
|  | Disagree | 59.8% | 74.4% |
|  | Neutral | 14.3% | 7.8% |
| Sometimes, it is necessary to plagiarize. | Agree | 36.8% | 31.1% |
|  | Disagree | 45.4% | 52.2% |
|  | Neutral | 17.8% | 16.7% |
